# Adherence to anti-seizure medications and associated factors among children with epilepsy at tertiary Hospital in Southwest Ethiopia: a cross-sectional study

**DOI:** 10.1186/s12883-022-02842-8

**Published:** 2022-08-23

**Authors:** Hawi Mohammed, Kemal Lemnuro, Teferi Mekonnen, Tsegaye Melaku

**Affiliations:** 1 Department of Pediatrics and Child Health, Dilla University, Dilla, Ethiopia; 2 School of Medicine, Werabe University, Werabe, Ethiopia; 3 Department of Pediatrics and Child Health, Jimma University, Jimma, Ethiopia; 4 School of Pharmacy, Institute of Heath, Jimma University, Jimma, Ethiopia

**Keywords:** Adherence, Attitude, Anti-seizure medications, Children, Epilepsy, Jimma medical center, Knowledge, Seizure control, South West Ethiopia

## Abstract

**Background:**

Childhood epilepsy causes a tremendous burden for the child, the family, society as well as the healthcare system. Adherence to anti-seizure medications (ASMs) is a key to treatment success. Poor adherence has been considered as one of the main causes of unsuccessful treatment for epilepsy and presents a potential ongoing challenge for achieving a key therapeutic goal of seizure control.

**Methods:**

A facility-based cross-sectional study design was conducted among children with epilepsy attending the Pediatrics neurology follow up clinic of Jimma Medical Center from June- 21 to September- 20, 2021. Data were collected by using a semi-structured pre-tested questionnaire. Epidata version 3.1 and SPSS version 26.0 were used for data entry and analysis respectively. Descriptive statistics and binary logistic regression analysis were employed. Adjusted odds ratios were used to ascertain effect sizes for any association between the dependent and associated variables while significance level at *p*-value of < 0.05 was determined using 95% confidence intervals.

**Result:**

A total of 170 children with epilepsy were included in this study. About 54.7% were male and 44.7% were in age range of 10–17 years. The overall adherence to anti-seizure medications was 54.1%. Those caregivers who were married [AOR = 7.46 (95% CI = 1.46, 38.20)], those children with controlled seizure status [AOR = 3.64 (95% CI = 1.51, 8.78)], those who got appropriate health care [AOR = 7.08(95% CI = 2.91, 17.24)], those caregivers who had good knowledge [AOR = 5.20(95% CI = 2.60,14.83)]; and positive attitude [AOR = 2.57 (95% CI = 1.06, 6.28)] towards epilepsy were significantly associated with adherence to anti-seizure medications.

**Conclusions:**

More than half of the children/adolescents having epilepsy were adherent to their anti-seizure medication(s). Children’s adherence to anti- seizure medications was influenced by current marital status of the parents/caregivers, controlled seizure status, getting appropriate healthcare in the hospital, caregiver’s knowledge; and attitude towards epilepsy. More efforts are required to scale up the provision of client-centered service (provision of appropriate health care delivery, focus on quality of treatment and providing health education/counseling to improve caregivers’ knowledge and attitude towards epilepsy) to improve children’s adherence status to their medication(s) and seizure control status.

## Introduction

Over 50 million people are affected by epilepsy worldwide of which 80% are in developing countries. Mortality is 2–3 times higher than that of the general population. The common causes of epilepsy-related deaths are sudden unexplained death in epilepsy (SUDEP) (2–18%) of all deaths in epilepsy, death due to status epilepticus (SE) (12.5%) and suicide (0–2%) [1]. Approximately 1 out of 150 children is diagnosed with epilepsy during the first 10 years of life, with the highest incidence rate observed during infancy [2].

A systematic analysis of epilepsy in sub-Saharan Africa showed that, active epilepsy was estimated to affect 4.4 million people in this region. The prevalence of active epilepsy per age group in the same region is 5.09/1000 and 5.98/1000 among children age 0–9 years old and 10–19 years old respectively [3].

Epilepsy symptoms can be successfully treated with one or more anti- seizure medications which can control seizures in 60–80% of patients with new onset epilepsy [4, 5]. The goal of treatment of epilepsy includes minimizing the risk of recurrent seizures and anti- seizure medications (ASMs) side effects, and maintaining normal psycho-social and educational/vocational adjustments [6].

Adherence to ASMs is key to treatment success, one of the main causes of unsuccessful drug treatment for epilepsy is poor adherence to prescribed medications [7, 8]. Medication adherence increase if the patients and families are involved in treatment choice as well as a mutual agreement between the clients and healthcare providers. Adherence to anti-seizure medications results in decrement of relapses, minimized frequency of seizures, decreased cost of health care, increased therapeutic benefits and better patient outcomes [9].

Despite the fact that there are significant treatment advances in pediatrics, improvements in medical technology to assess adherence behaviors and increased focus on adherence in research and clinical practice, non-adherence rates across all pediatric chronic illnesses is quite high, 50–75% [9]. According to WHO’s adherence to long-term therapies, adherence to anti- seizure medications in children with epilepsy ranges from 25 to 75% [10]. Specifically; the prevalence rate of adherence to ASMs was 55% in South Africa in 2016 [11], 55.2% in Nigeria in 2020 [12], 79.5% in Uganda in 2014 [13], 61.7% in Saudi Arabia in 2015 [14], 29% in Indian subcontinent in 2018 [15], 21.3% in western China in 2020 [16]; and 42% in USA in 2011 [17].

A study done in Pakistan (2018) on 120 children with epilepsy on ASMs for at least 1 month showed that 70(58%) of children had suboptimal ASM adherence and 50(42%) of them had satisfactory adherence according to self or parental report. In this study, ASM prescription patterns, adverse effects, availability of ASMs, seizure control, treating physician’s counseling, financial constraints; and parental/caregiver’s education were more significantly associated with drug non-adherence in these communities [18].

Another study done in Turkey in 2020 on 226 children and adolescents with epilepsy and their primary caregivers, the overall prevalence of complete drug adherence among the patients was 47.3%. The main reasons of non-adherence to ASMs were forgetting to take medication (33.6%) and the difficulties in adhering to treatment (24.3%). In contrast to above studies, age and caregivers’ health literacy knowledge were found to be significantly associated with adherence in this study. Patients in the 0 to 5 years age group were more likely to have full drug adherence than were those in the 12 to 18 years age group [19].

On the other hand, a study done on 112 children aged 2-14 year at Queen Rania AL-Abdullah Children Hospital in 2015 showed that 79.5% of the patients display some adherence to anti-seizure medication, while 20.5% of the patients do not display adherence to their anti- seizure medication. 23 patients (20.5%) have low adherence, 56 patients (50%) have medium adherence, and 33 patients (29.4%) have high adherence. In this study, parents’ forgetfulness to give medications to their children (52.2%), fear of medication side effects (33.5%), and being improved and seizure free for a period (30.4%) were significantly associated with non-adherence [20].

Seizure control status may affect patients’ adherence to ASMs as it will increase treatment satisfaction; a cross-sectional observational study was conducted among 253 patients with epilepsy in Bangladesh indicate that adherence was 38.7% and associated with well seizure control. Among ASM adherent patients (*n* = 98), 70(82.4%) had controlled seizure status [21].

Treatment of epilepsy and measurement of adherence to ASM in children has been a major challenge because of the chronicity of the disease and the dependency of children on their parents/caregivers. Further, low adherence and poor seizure control will adversely affect socio-behavioral and cognitive function of children which lead to poor school performance. The factors that affect adherence to ASMs reported by various studies are not similar and there is a lack of information regarding the prevalence and associated factors of adherence to ASMs in the study area and Ethiopia in general. In addition, previous few studies done in Ethiopia focused on adult patients at the community level and focus of health policy of Ministry of health-Ethiopia was on prevention, even though more recently chronic illness including epilepsy gained more attention. Different socio-demographic, clinical and treatment, health services, parental knowledge and attitude, and psychosocial related factors may contribute to non-adherence to ASMs. Therefore, taking into account the existing problem under study, which is a critical and major public health problem and having limited information because of lack of published study on the issue of adherence to ASMs in the study area farther strengthen the importance of this study.

## Methods and materials

### Study area and period

This study was conducted from June 21- Sep 20, 2021, in Jimma Medical Centre (JMC), Jimma, South West Ethiopia. The center is one of the oldest public hospitals in the country located in Jimma town of Oromia Regional State, Ethiopia. JMC gives services for an estimated 20 million people from Jimma zone and is a referral centre for regions of South Western part of Ethiopia. It also serves as a teaching hospital for several undergraduate and post graduate programs for medicine and health sciences students of Jimma University.

### Study design, source population and study population

A facility based cross- sectional study design was employed on children with epilepsy. All children having epilepsy on follow- up attending Paediatrics neurology clinic of JMC were source populations. Children between 6 month- < 18 years of age having epilepsy visiting JMC Paediatrics neurology clinic and those who fulfilled the eligibility criteria were involved in this study.

### Sample size determination

The required sample size for this study was determined by using single population proportion estimation formula and considering the following assumptions; Study done in Joe, Nigeria showed that the proportion of adherence to ASMs was around 55.2% with a 95% of the confidence interval and 5% of margin of error was taken. Accordingly, the calculated sample size was 380.

Since the source population is < 10,000; the final sample size was determined by applying the finite population correction formula and adding 5% non-response rate. Accordingly, the calculated final sample size became 170 children/adolescents with epilepsy.

### Sampling technique and procedure

According to the three consecutive months Health Management Information System (HMIS) activity report of JMC; the 3 months average visit/load at paediatrics neurology clinic was around 280 children with epilepsy; hence by using this data and calculated sample size which was 170; all parents/caregivers of children and/or adolescents with epilepsy who fulfilled the inclusion criteria, consented (parents/caregivers) and assented (adolescents) to participate in the study were interviewed at k^th^ interval of 1.6(~ 1)(280/170); then patients were interviewed consecutively until required sample size was obtained at their exit from neurology clinic.

### Data collection tools and procedures

Data were collected by two registered BSC pharmacists and supervised by principal investigator by using pre-tested interviewer administered semi-structured questionnaire which was developed by compiling a number of questions adapted from similar study materials and review of relevant literatures [22–24] to address the objectives of this study. The questionnaires were prepared in six sections: section-I was about parents’/caregivers’ socio-demographic and economic related factors; section-II was about children’s socio-demographic, clinical and treatment related factors; section-III was about health facility/service related factors; secion-IV was about to assess parental knowledge about epilepsy; section-V was about to assess parental attitude towards epilepsy; and secion-VI was about ASM adherence status. The questionnaires were prepared in the English language and then translated to the local languages: Amharic & Afaan Oromo by language experts and re-translated back to English by experts to check consistency before conducting data collection.

The study participants were given an orientation on the details concerning participation in the study and informed verbal consent was obtained from each eligible study participant. The eligible participants were interviewed at their exit from the neurology follow-up clinic. The diagnosis of each child/adolescent including co-morbidities, types of ASM(s) he/she was taking and some of the ASM side effects were retrieved from charts at time of interview. The principal investigator supervised closely the overall data collection activities on a daily basis.

### Operational definitions and variable measurements

Adherence to ASMs measured by eight-item Morisky Medication Adherence Scale (MMAS) [22] that is widely used to measure adherence. Items 1–7 are yes/no questions, in which a “no” answer receives a score of 1 and a “yes” answer receives a score of 0, except for item 5, which is reverse scored. Item 8 is measured on a five-point scale. The responses “never”, “once in a while”, “sometimes”, “usually”, and “all the time” are scored, 1, 0.75, 0.50, 0.25, and 0 respectively and the total score ranges from 0 to 8.

- Low adherence/non-adherence:- Patients scores < 6 of 8 items MMAS.
- Medium adherence: - Patients scores 6–7 of 8 items MMAS.
- High adherence: - Patients scores 8 of 8 items MMAS.

Overall adherence: - Dichotomized as adherent and non-adherent. In this study, individuals in category of medium and high adherence were taken as ‘adherent’ and low adherence as ‘non-adherent.’

### Data management and quality control

To keep the uniformity of the data collection process; data collectors were trained for 2 days on the objective of the study, method of data collection and interview technique. A pre-test was conducted on 5% (9) children having epilepsy in the pediatrics neurology clinic, JMC 1 week before real data collection to assess its clarity, completeness and consistency and then necessary adjustment was done to the tools.

Data were checked for completeness, accuracy and consistency by principal investigator on a daily basis. Double entry of data for checking errors was performed to assure quality of data before analysis.

### Data processing & analysis

The data on the questionnaire were entered into Epidata manager version 3.1 and exported to SPSS version 26.0. The data were edited and cleaned for inconsistencies, explored to check outliers and missing data.

Descriptive statistics was calculated for socio-demographic and economic status of the participants, child’s health related factors (clinical and treatment profiles) and health facility related factors. Variables for knowledge about epilepsy, attitude towards epilepsy, seizure control; and level of adherence were computed using variables recoding. Bivariate analysis was performed to select variables for multivariate analysis. Hence variables with a *p*-value ≤0.25 in the bivariate analysis were taken as candidates for multivariable analysis. Finally, multivariable logistic regression analysis was performed to identify the independent predictors of Adherence to ASMs. Variables with a *p*-value of < 0.05 in multivariable logistic regressions were taken as statistically significant predictors for adherence and OR with its 95% CI was used to show the degree of association between the independent and the outcome variable. Results were reported as percentages (frequency) for categorical variables and findings were summarized and presented in the form of tables and interpreted in the line of its objective with narration.

## Results

### Socio-demographic and economic characteristics of participants/caregivers

A total of 170 children/adolescents having epilepsy on follow- up were involved in this study. Nearly nine from ten (150, 88.2%) of caregivers were father (44.1%) and mother (44.1%). The age of most (70, 41.2%) of parents/caregivers was between 18 and 35 years and more than half (98, 57.6%) of them were from rural area. About 132(77.5%) of parents/caregivers were married and 66(38.8%) of them cannot read and write. Nearly half (84, 49.4%) of parents/caregivers were farmers and more than half (93, 54.7%) of their monthly income was 1500–3500 Ethiopian birr (Table 1).

**Table 1:** Socio-demographic and economic related characteristics of study participants/caregivers

Variables	Category	Frequency	Percentage (%)
	**Caregivers’ background**		
Primary caregiver (Caregiver’s identity)	Father	75	44.1
	Mother	75	44.1
	Sibling	9	5.3
	Grandparents	8	4.7
	Uncle/Aunt	3	1.8
Age of caregiver (years)	18–35	70	41.2
	35–45	53	31.2
	≥ 45	47	27.6
Residence	Urban	72	42.4
	Rural	98	57.6
Current marital status of caregivers	Single	11	6.5
	Married	132	77.5
	Separated	7	4.1
	Divorced	9	5.3
	Widowed/Widower	11	6.5
Educational status of the caregivers	Cannot read & write	66	38.8
	Can read and write	29	17.1
	Primary school	23	13.5
	Secondary school	36	21.2
	College/University	16	9.4
Occupational status of the caregivers	House wife	28	16.5
	Farmer	84	49.4
	Merchant	33	19.4
	Government employee	20	11.7
	Daily laborer	4	2.4
	Others^a^	1	0.6
Family size	≤ 5	93	54.7
	> 5	77	45.3
Average monthly income	< 1500 ETB^b^	37	21.8
	1500–3500 ETB	93	54.7
	> 3500 ETB	40	23.5
	**Children’s background**		
Age of the child (years)	< 1	2	1.2
	1–5	39	22.9
	6–10	53	31.2
	10–17	76	44.7
Sex of the child	Male	93	54.7
	Female	77	45.3
Educational status of the child	Pre-school	61	35.9
	Primary school	73	42.9
	High school	4	2.4
	Not attend school	32	18.8

^a^ Student, ^b^ Ethiopian Birr

More than half (93, 54.7%) of the children with epilepsy were male, most (76, 44.7%) of them were adolescents(10–17 year) & 32 (32/109, 29.4%) of them didn’t attend school (Table 1).

### Child’s health related factors (clinical and treatmentprofiles)

Among 170 of the study participants; 104(61.2%) of them were diagnosed with generalized seizure, 10(5.9%) of them had family history of epilepsy. Around 106(62.4%) of study participants were on treatment for more than 2 years and 108(63.5%) of them were on monotherapy. Mostly prescribed ASM was phenytoin only, 82(48.2%). 44(25.9%) of the children were ever stopped/missed their ASM doses since starting of therapy; main reason was forgetfulness, 31(70.5%). More than one-fourth, 46(27.1%) of the study participants reported some adverse effects with ASMs, among these more than half (24, 52%) of them were reporting drowsiness **(**Table 2**).**

**Table 2:** Health related factors (clinical and treatment profiles) among study participants

Variables	Category	Frequency	Percentage (%)
Main seizure type	Focal seizure	30	17.6
	Generalized seizure	104	61.2
	Unclassified seizures	36	21.2
Duration since diagnosis (in years)	≤ 2	58	34.2
	> 2	112	65.8
Duration on treatment (in months)	6–12	34	20.0
	13–24	30	17.6
	> 24	106	62.4
Family history of	Yes	10	5.9
seizure/epilepsy	No	155	91.2
	I am not sure	5	2.9
Mode of therapy used	Mono-therapy	108	63.5
	Dual therapy	61	35.9
	Triple therapy	1	0.6
ASMs prescribed	Phenobarbitone only	24	14.1
	Phenytoin only	82	48.2
	Valproate only	2	1.2
	Phenytoin and Phenobarbitone	52	30.6
	Phenytoin and Valproate	4	2.4
	Phenobarbitone and carbamazepine	5	2.9
	Phenobarbitone, phenytoin and Valproate	1	0.6
Stopped/ missed ASMs doses	Yes	44	25.9
	No	126	74.1
Main reasons for stopping or for not taking the tablets regularly (***n*** **= 44**)	Forgetfulness	31	70.5
	Fear of side effects	13	29.5
	Feeling better	7	15.9
	Run out of drug/Did not get adequate tablets from the hospital	16	36.4
	Child refuses to take drugs	9	20.5
	Financial constraints	9	20.5
	Others^a^	6	13.6
Adverse effects to ASMs	Yes	46	27.1
	No	118	69.4
	I do not know	6	3.5
Types of ASMs adverse effects (***n*** **= 46**)	Behavioral abnormality	9	19.6
	Gum hyperplasia/swelling	10	21.7
	Skin rash	2	4.3
	Drowsiness	24	52.2
	Fatigue	11	23.9
	Decreased concentration	6	13.0
	Constipation	2	4.3
	Others^b^	2	4.3
Does the child have other additional diagnosis?	Yes	9	5.3
	No	161	94.7
Other comorbid conditions (***n*** **= 9**)	Cerebral palsy	6	66.7
	Developmental delay	1	11.1
	Microcephaly	1	11.1
	T1DM^c^	1	11.1
Use of other ancillary medication(s)	Yes	1	0.6
	No	169	99.4
Use of herbal/traditional medicine as additionally	Yes	9	5.3
	No	161	94.7
Seizure frequencies in the last 3 months (Seizure control status)	No seizure (Controlled seizure)	92	54.1
	≥ 1 episode (Un-controlled seizure)	78	45.9

^a^ COVID-19 (4), Mourn (2)^b^ Dyspepsia (Epigastric discomfort)^c^ Type-one diabetes mellitus

### Health facility/ service related factors

Among the study participants, 73(42.9%) of them travel more than 20kms to arrive JMC and 119(70%) of them were using public transport. More than half (100, 58.8%) of study participants received counseling about epilepsy and/or ASMs sometime during follow up visit; 84(84%) of them were counseled about ‘importance of ASMs’ and 79(46.5%) of them get ASMs free of charge (Table 3).

**Table 3:** Health facility/ service related factors among study participants

Variables	Category	Frequency	Percentage (%)
Distance from home to hospital	≤ 10 km	68	40.0
	10–20 km	29	17.1
	> 20 km	73	42.9
Means of transport to the hospital	On foot	13	7.6
	Public transport	119	70.0
	On foot then public transport	38	22.4
Time taken from home to hospital	< 30 minutes	41	24.1
	30–60 minutes	55	32.4
	> 60 minutes	74	43.5
Health education and/or advice about epilepsy and ASMs	Yes	100	58.8
	No	70	41.2
Type of counselling/advice given about ASMs? (***n*** **= 100**)	Importance of ASMs ^a^ tablets	84	84.0
	Method of use	50	50.0
	Side effects of the drug(s)	42	42.0
	Not to miss dose and appointment	43	43.0
ASM(s) refill (Way of getting the medication(s))	By cash/payment	12	7.0
	By CBHI^b^	7	4.1
	Free of charge	79	46.5
	Free sometimes cash	72	42.4
Problems faced in the hospital during health care delivery (inappropriate health care)	Yes	75	44.1
	No	95	55.9
Type(s) of problem(s) faced in the hospital during health care delivery(*n* = 75)	Shortage of ASMs	68	90.7
	Long waiting time	18	24
	Poor communication from the staffs	13	17.3

^a^ Anti-seizure medications ^b^ Community based health insurance

### Parents/caregivers knowledge and attitude about and towards epilepsy respectively

Among the study participants; more than half (97, 57.1%) of them were found to have overall good knowledge about epilepsy and 95(55.9%) of them were having positive attitude towards epilepsy (Table 4).

**Table 4:** Overall knowledge and attitude of parents/caregivers about and towards epilepsy

Variables	Category	Frequency	Percentage (%)
Parental knowledge about epilepsy	Poor knowledge	73	42.9
	Good knowledge	97	57.1
Parental attitude towards epilepsy	Negative Attitude	75	44.1
	Positive Attitude	95	55.9

### Adherence status to ASMs of the study participants

Among 170 study participants, more than half (54.1%) of them were found to be adherent to their ASM(s) (Table 5).

**Table 5:** Overall adherence status to ASMs among study participants

Variables	Category	Frequency	Percentage (%)
Adherence status to ASM(s)^a^	High adherence to ASM(s)	18	10.6
	Moderate adherence to ASM(s)	74	43.5
	**Overall adherence to ASM(s)**	**92**	**54.1**
	Non-adherent to ASM(s)	78	45.9

^a^Anti-seizure medication

### Predictor variables of adherence to ASMs

After bivariate logistic analysis, multivariable logistic regression analysis was done to calculate odds ratios and corresponding 95% confidence intervals for the predictors of adherence to ASMs. Those caregivers who were married [AOR = 7.46 (95% CI = 1.46,38.20)], those children with controlled seizure status [AOR = 3.64 (95% CI =1.51, 8.78)], those who got appropriate health care [AOR = 7.08 (95% CI = 2.91,17.24)], children of those caregivers who had good knowledge [AOR = 6.20 (95% CI = 2.60, 14.83)] and positive attitude [AOR = 2.57 (95% CI = 1.06, 6.28)] were significantly associated with adherence to ASMs (Table 6).

**Table 6:** Bivariate and multiple logistic regression model factors associated with adherence to ASMs among study participants

Variables	Category	ASMs Adherence status		COR (95%CI)	***P***-value*	AOR (95%CI)	***P***-value*
		Non Adherent	Adherent				
Current marital status of caregiver	Single	7	4	0.66 (0.11,4 .01)	0.648	1.73 (0.16, 18.85)	0.655
	Married	47	85	2.69 (0.75, 9.66)	0.129*	7.46 (1.46, 38.20)	0.016*
	Separated	6	1	0.70 (0.90, 5.43)	0.733	0.50 (0.05, 5.32)	0.561
	Divorced	8	1	.88 (0.14, 5.58)	0.888	1.67 (0.17, 16.83)	0.665
	Widowed/Widower	10	1	1		1	
Educational status of the child	Pre-school	26	35	1.97 (0.83, 4.69)	0.127*	1.85 (0.53, 6.48)	0.333
	Primary school	30	43	2.10 (0.90, 4.88)	0.087*	2.92 (0.89, 9.57)	0.077
	High school	3	1	0.49 (0.05, 5.22)	0.552	0.61 (0.01, 79.49)	0.844
	Not attend school	19	13	1		1	
Seizure control status	No seizure (Controlled)	29	63	3.67 (1.94, 6.93)	0.000*	3.64 (1.51, 8.78)	0.004*
	≥ 1 episode (Uncontrolled)	49	29	1		1	
ASMs ^a^ refill (Way of getting the medications)	Cash/payment	5	7	1.75 (0.51, 6.04)	0.376	2.02 (0.37, 11.12)	0.420
	CBHI^b^	5	2	0.50 (0.09, 2.75)	0.425	0.32 (0.03, 3.80)	0.370
	Free of charge	28	51	2.28 (1.18, 4.38)	0.014*	1.33 (0.52, 3.31)	0.544
	Free sometimes cash	40	32	1		1	
Problems faced in the hospital (Inappropriate healthcare)	No	20	75	0.15 (0.08, 0.29)	0.000*	7.08 (2.91, 17.24)	0.000*
	Yes	58	17	1		1	
Parental knowledge about epilepsy	Good knowledge	21	76	0.13 (0.10, 0.26)	0.000*	6.20 (2.60, 14.83)	0.000*
	Poor knowledge	57	16	1		1	
Parental attitude towards epilepsy	Positive Attitude	56	36	0.93 (0.21, 0.73)	0.003*	2.57 (1.06, 6.28)	0.038*
	Negative Attitude	22	56	1	0.648	1	

*P-value < 0.05, ^a^ Anti-seizure medications, ^b^ Community Based Health Insurance

## Discussion

Adherence to anti-seizure medications is a key to treatment success, one of the main causes of unsuccessful drug treatment is poor adherence to prescribed medications [7, 8]. Adherence to anti-seizure medications results in decrement of relapses, minimized frequency of seizures, decreased cost of healthcare, increased therapeutic benefits and better patient outcomes [9]. This study was conducted to assess the prevalence of ASM adherence and associated factors among children/adolescents having epilepsy on follow- up in JMC.

The adherence status to ASMs in this study was 54.1% and the rest(45.9%) of the study participants were non-adherent to ASMs. The prevalence of adherence to ASMs in children having epilepsy is similar to the study done in South Africa(55%) in 2016 [11], Joe, Nigeria(55.2%) in 2019 [12] and Turkey (47.3%) in 2020 [19], this might be because of similar socio-demographic status of the study population participated and use of similar scales (MMAS-8) (in Nigerian & Turkish study) to assess adherence in these studies.

However, the prevalence of adherence to ASMs in our study was significantly lower than the study done in 94 children in the Kingdom of Saudi Arabia(61.7%) in 2015 [14]. The possible justification for the disparities between these studies might be due to different demographic status including socio-economic status and higher literacy ratio, lower study population(*N* = 94) and use of earlier scale (MMAS-4) to assess adherence in this study; and also lower than the study done in Uganda(79.5%) in 2014 [13], this difference might be because of higher literacy ratio and use of different scales to assess adherence to ASM (self report- focus group discussion).

The prevalence of adherence to ASMs in this study was higher than that of studies done in Pakistan(42%) in 2018 [18], USA(42%) in 2011 [17], Bangladesh (38.8%) in 2015 [21], Indian subcontinent(29%) in 2018 [15] and Western China(27.3%) in 2020 [16]; these differences might be due to long duration of studies (USA and Bangladesh) and use of different adherence measuring scales(e.g. Electronic method to measure adherence in study done in USA) and different definitions of adherence to ASMs(e.g. moderate and low adherence were considered as ‘poor adherence’ in study done in Western China and Indian Subcontinent).

There were different factors contributing to the adherence to prescribed anti-seizure medications. The current study identified marital status of the caregivers; i.e. being married caregiver(s)/parent(s) was associated with the adherence. This might be due to stable and full family support (similar with the study done in KSA, 2015). Others associated with adherence to ASMs include children/adolescents whose seizure was controlled might be due to treatment satisfaction and frequency of seizure will increase in those who were non- adherent to thier medications (in study done in KSA, 2015). Parents/caregivers who recieved appropriate health care in the hospital during follow- up visit were more than 6 times adherent to their ASMs; this was due to smooth and friendly communication with hospital staff (which was similar with the study done in KSA, 2015) and also might be due to recieving of appropriate health/drug information from medical staff (similar with the study done in W. China, 2020); those parents/caregivers whose knowledge about epilepsy was good (about 6 times) and positive attitude (about 3 times) towards epilepsy were more adherent to ASMs (increased awareness about epilepsy & ASMs might contribute, supported by study done in Turkey, 2020).

In a study done in South Africa [11], long duration of therapy and medications (Oxcarbamazepine, Valproic acid and Phenytoin) were significantly associated with adherence to ASMs; the age of the patients, type of epilepsy, total household income and source of drug information in Western China [16]; monotherapy, good family support & lower frequency of seizures (similar to our study) in KSA [14] were significantly associated with adherence to ASMs.

Unlike our study, higher family socioeconomic status was significantly associated with ASM adherence in a study done in USA [17]; low socioeconomic status, multiple drug intake & long duration of therapy were significantly associated with ASM non-adherence in Joe, Nigeria [12].

Seizure control status is a key to adherence to ASMs which helps patients stick to their medications. In this study, seizure control status was significantly associated with adherence. Our finding was similar to the study done in Indian Subcontinent (seizure control = 51.78%) in which one or more seizure episodes in the past 3 months (un-controlled seizure) adversely affected ASM adherence [15] and higher than the study done in Bangladesh (33.6% seizure control status) which was significantly associated with adherence to ASMs [21]. In contrast to our study, age of the child (0–5 years, because drugs are given by the patients/caregivers, while adolescents usually manage their own treatment) was significantly associated with ASM adherence in a study done in Turkey [19].

Being on multiple medications can lead to non-adherence in many ways; including increased medication cost, pill burden and more adverse drug reactions. Pill burden is particularly a problem in children and adolescents and refusal to take drugs is worse with increasing number of medications. Poly-therapy is more likely to be associated with drug toxicity [12]. Nearly two third (108, 63.5%) of our study participants were on mono-therapy (similar to study done in Nigeria, 59.3%) [12], from this ‘Phenytoin only’ accounted for nearly half (82, 48.2%) of cases; from those (those on mono-therapy) more than half of them were adherent to their medication(s) but mode of therapy was not associated with adherence status to ASMs in this study.

Parents’/caregivers’ knowledge about epilepsy was similar to the study done in Ethiopia (North Shoa, 2018) (56.4%) [23]; similar cultural, economic, and sociodemographic features might contribute. Our finding was significantly associated with adherence to ASMs; similar with the study done in Turkey (caregivers’ health literacy knowledge was significantly associated with ASMs adherence,) [19]; and our finding was significantly higher in comparison to the study done in India (10/60, 16.7%) [25], this difference might be due to small sample size(*N* = 60).

Parents’/caregivers’ positive attitude towards epilepsy was significantly associated with adherence status to ASMs which was comparable with the study done in India (55%) [25] and it was comparable with the study done in Ethiopia (North Shoa, 2018)(58.7%) [23].

### Limitations

Hence the actual prevalence of adherence to ASM(s) may be even lower because we used parent/caregiver and/or adolescent-report to assess adherence status which may be subjected to recall bias as some may feel pressured to give acceptance responses to gain positive reaction from data collectors/health care workers.

The research was done in single institution which may not help to generalize the findings of this study for regional or country level and samples were taken from hospital which might not be representative as many patients may not come to hospital despite their illness for different reasons.

Further, many patients were not had EEG for the diagnosis of epilepsy; thus diagnosis of epilepsy and initiation of ASMs was based on clinical evidences.

Lastly, sample size was drawn based on study done in Nigeria which is far from our study area (Ethiopia).

## Conclusions

More than half of the children/adolescents having epilepsy were adherent to their anti-seizure medication(s). Children’s adherence to anti- seizure medications was influenced by current marital status of the parents/caregivers, controlled seizure status, getting appropriate healthcare in the hospital, caregiver’s knowledge; and attitude towards epilepsy. More efforts are required to scale up the provision of client-centered service (provision of appropriate health care delivery, focus on quality of treatment and providing health education/counseling to improve caregivers’ knowledge and attitude towards epilepsy) to improve children’s adherence status to their medication(s) and seizure control status.

## Data Availability

All materials and data are available from the corresponding author without any restriction.

## Abbreviations

ASM: Anti-seizure medication
CBHI: Community based health insurance
CP: Cerebral palsy
DC: Data collector
ETB: Ethiopian birr
HMIS: Health management information system
ILAE: International league against epilepsy
IRB: Institutional review board
JMC: Jimma medical center
KSA: Kingdom of Saudi Arabia
OR: Odds ratio
SE: Status epilepticus
SSA: Sub-saharan Africa
